# Cost-effectiveness of bringing a nurse into an Italian genetic day clinic: a before and after study

**DOI:** 10.1186/s12913-023-10238-8

**Published:** 2023-11-20

**Authors:** Marina Mordenti, Morena Tremosini, Manuela Locatelli, Maria Gnoli, Cristiana Forni, Elena Pedrini, Manila Boarini, Luca Sangiorgi

**Affiliations:** 1 Department of Rare Skeletal Disorders - IRCCS Istituto Ortopedico Rizzoli, Bologna, Italy; 2 Nursing and Allied Profession Research Unit - IRCCS Istituto Ortopedico Rizzoli, Bologna, Italy

**Keywords:** Genetic nurse, Health services research, Cost effectiveness research, Quality of care, Rare skeletal diseases

## Abstract

**Background:**

Only a few studies explore the role of nurses in genetic counselling and genetic health care, and none of them is related to orphan diseases. In addition, few studies address the issue of finding variables that might affect the economy of a service or perform a cost-effectiveness analysis of a having genetic nurse at a unit.

**Methods:**

A multidisciplinary panel of experts working in the hospital was set up to identify sensitive indicators and remove confounding variables. This panel evaluated efficiency and effectiveness indicators and drafted a questionnaire to estimate patient perception of the quality of the service. Data were captured from different sources, including the hospital patient database and a web-accessible platform for data collection. More than 600 clinical evaluations of 400 patients were considered, and economic parameters were studied by applying Porter’s Time-Driven Activity-Based Costing methodology to evaluate costs and outcomes. Additionally, an anonymous, semi-structured, paper-and-pencil interview questionnaire was given to patients at their periodic follow-ups.

**Results:**

The results showed an increase in the quality of patient management, more accurate data capturing, and higher quality ambulatory care. In fact, approximately 70% of the respondents reported positive changes. In addition, a parallel economic analysis explored indicators influencing economic impact, and outcomes showed positive results with the quality of outcomes improving more compared to the increase in costs.

**Conclusions:**

The variety of evaluated issues highlighted that having a nurse in a genetic service and at day clinic activities resulted in better access, better scheduling, more satisfaction, and proved to be a cost-effective solution for patients affected by rare diseases.

**Supplementary Information:**

The online version contains supplementary material available at 10.1186/s12913-023-10238-8.

## Background

Only a few studies explore the role of nurses in genetic counselling and genetic healthcare pathways [1, 2]. None of them is relative to orphan diseases. They usually concern nursing in oncology. Some studies stress the importance of nurses in genetic and genomic healthcare assistance [3]. Moreover, many studies investigate the educational needs, the subsequent training, and the derived knowledge of nurses and midwives [4–6]. Fewer studies address the issue of finding variables that might affect the economy of a service or analyze the cost-effectiveness of having genetic nurses in a unit [7, 8]. Some studies describe the effectiveness of having a nurse at a service under very specific conditions, for example by evaluating the nurse as a substitute for a general practitioner in an after-hours primary care setting [9]. Carter and Chochinov [10] measured the impact of nurse practitioners in emergency departments, but this study was performed in genetic units with expertise in cancer. However, only a few studies give a historical perspective of the development of genetic nurse specialists [11]. Conversely, this study is aimed at surveying and measuring changes resulting from bringing a nurse into an existing medical genetics service that caters to orphan patients, healthy carriers, and their families, regardless of specific role or genetic background. According to the literature, nurses are front-line healthcare professionals for patients affected by hereditary disorders, especially in cancer-related diseases [12], and the interaction between nurse and patient is a key element for high-quality care [13]. In addition, nursing care is intended to provide holistic, family, and patient-centred care that aligns with genetic counselling [14], and nurses are frequently involved as case managers in research activities [15]. In the US and many European countries, genetics nurses are often part of a multidisciplinary team in genetics units. In Italy, they are seldom integrated in medical genetics services. Although the Italian Society of Human Genetics recommended having a nurse in clinical genetics units, only a few centres have fulfilled this requirement at present (https://sigu.net/wp-content/uploads/2021/01/2317-STANDARD-SIGUCERT-STRUTTURE-CLINICHE-DI-GENETICA-MEDICA_Rev2019.pdf).

The genetics day clinic (GDC) proposing the study has been active at the hospital since 2003. The GDC works on rare hereditary dysplasias and neoplastic syndromes involving the musculoskeletal apparatus by offering genetic counselling, molecular analysis, and orthopaedic/physiatrist follow-up for patients and their relatives. At the beginning, the pool of professionals involved in patient management included a medical geneticist, an orthopaedic specialist, and a psychologist, all involved in patient care and genetic counselling. A need to improve the treatment of the disease and patient management was identified, and this led to bringing a nurse into the clinical practice alongside the existing multidisciplinary staff, as per literature [16].

Considering the complexity of the nurse's role (organizing the patient’s trajectory through the healthcare system, understand the patient’s feelings, promoting health, participating in research activities, etc.), no proper evaluation tools were individuated in the literature. In fact, most of the studies are intended to assess the nurse’s perception and involvement [17]. In addition, due to the small number of genetics services that have hired nurses with specific expertise in genetics, few studies on this topic have been published, [18] even if recent literature highlights the impact of their role [19]. At present, in Italy, there are no studies that assess the impact of having a nurse in this field. Therefore, the present study identifies and proposed adequate indicators (variables) and assesses the impact of this professional figure, based on specific Italian accreditation requirements and literature indications [20, 21]. A non-secondary aspect is the economic impact of the implementation: a cost-effectiveness analysis, evaluating a set of variables that might affect the economy of the medical genetics service was performed, leading to a clearer picture of all the aspects related to bringing a nursing unit into the genetics service. A Porter’s Time-Driven Activity-Based Costing methodology [22, 23] was applied to evaluate the organizational modification to the present system to set up a matrix that clearly shows possible value improvements and their economic sustainability.

## Methods

This study aimed at surveying and measuring changes resulting from bringing a nurse into an existing medical genetics clinic, considering patient perception of service quality, quantifying efficiency and effectiveness, and evaluating the economic impact. A multidisciplinary panel of experts working in the hospital (composed of two medical geneticists, two nurses, two researchers, one psychologist, two administrative professionals, one economic research analyst, and one quality service reference) was set up before and after hiring the nurse (in 2017) to identify sensitive indicators and remove any confounding variables. The panel evaluated efficiency and effectiveness indicators as well as indicators for measuring quality of data comparison between 2017 and 2019. In fact, to clean up the data and increase the reliability of the results, 2018 was not considered a period of observation because it was primarily dedicated to the nurses’ integration and training, in the new organizational model. In addition, the multidisciplinary panel drafted a short self-reported questionnaire to estimate patient perception of the quality of the medical genetics service. In this study, administrative information and disease and examination data came from two separate IT tools: the hospital patient database and a web-accessible platform in use at the GDC. The present study was approved by the institutional independent review committee (prot. N. 30394).

### Indicators of efficiency and effectiveness

The indicators of efficacy and effectiveness were defined according to the literature and national and regional indications. To assess the impact of the nurse in the process, the expert panel quantified its efficiency, considering the number of medical visits, diseases treated and blood samples collected. These variables were selected because they represent valuable and reliable indicators to show the changes before and after hiring the nurse, according to national and regional specific accreditation requirements [20].

Since the effectiveness represents the degree to which a patient receives the right care at the right time in the right place, leading to the best outcome, [21] to measure it, several indicators were included, comprising process and accessibility aspects — part of the appropriateness of treatment in national requirements. The selected indicators were: number of visits (broken down into first evaluation and follow-up), appropriateness of visits, considering the total number of children evaluated vs. children clinically evaluated by paediatric orthopaedics and the number of patients affected by osteogenesis imperfecta vs. number of osteogenesis imperfecta patients evaluated by a clinician with expertise in this disease, the number of cases without definite diagnosis, the number of blood samples collected from patients without final diagnosis, wait time for the first clinical evaluation, the suitable collection of clinical information/clinical records in the medical genetics service archive, and the number of projects submitted and accepted by the ethics committee. The effectiveness variables were individuated to measure the ability to achieve the objectives with the lowest possible resource allocation (time, costs, procedures on patients, etc.).

### Patient’s perception of service quality

The expert panel designed a short survey organized as a semi-structured interview to be submitted to patients at their periodic follow-ups referring to the GDC from 2017 until the end of 2019 to evaluate patient satisfaction. This was an anonymous, paper-and-pencil questionnaire submitted by a professional from a department at the institute (other than the GDC) and returned at the end of the same day. The patient questionnaire was divided into two sections: a set of brief, self-completion questions (identifying positive and negative changes in clinical and care pathways, especially information provided, wait times, ambulatory care) to investigate quantitative data and some open-ended exploratory questions to give patients the opportunity to describe in detail their own experiences, collecting additional qualitative information (see Supplementary Material 1 – Italian Survey for patient's perception of service quality).

### Visit dataset

The dataset evaluated was composed of all the visits carried out at the GDC, including but not limited to orthopaedic evaluation, genetic counselling, and reproductive counselling, divided into two groups: Group 1 spanned from January 1, 2017 to December 31, 2017 and Group 2 spanned from January 1, 2019 to December 31, 2019. We collected information on 604 clinical evaluations of 426 patients in the two periods (some patients were visited twice a year).

### Statistical analysis

All continuous data were expressed as mean and standard deviation of the mean, and categorical variables were expressed as frequency and percentages. The Kolmogorov–Smirnov test was performed to test the normality of continuous variables, and the Levene Test was used to assess homoscedasticity. The ANOVA test was performed to assess the differences in continuous, normally distributed, and homoscedastic data between groups, and the Mann Whitney test was used otherwise. The ANOVA test, followed by a Scheffé post-hoc pairwise comparison, was also used to assess the differences in continuous, normally distributed, and homoscedastic data between groups. The Kruskal–Wallis test, followed by the Mann–Whitney U test with the Bonferroni correction for multiple comparison was used otherwise. The general linear model (GLM) was also used as a multivariate analysis to assess the influence of potential predictive factors on wait times. Fisher’s test was performed to investigate the relationships between dichotomous variables. Pearson’s chi-squared test, evaluated by exact methods for small samples, was performed to investigate the relationships between grouping variables. For all tests, p < 0.05 was considered significant. All statistical analyses were performed using SPSS v.19.0 (IBM Corp., Armonk, NY, USA).

### Cost-effectiveness analysis

To evaluate the economic impact of bringing a nurse into the service, the value defined as “health outcome per euro spent” was measured. The evaluation followed the methodology proposed by Porter, revised for this specific situation, to set up a matrix that clearly shows value improvements or deteriorations as a trade-off between outcomes and costs. Value is defined as outcomes divided by costs. Outcome, the numerator in the value equation, are inherently condition specific and multidimensional. The assessment of outcomes used a six-step process:

1. Creating a hierarchy for measuring outcome at six sub-levels;
2. Mapping the basic parameters on the logs;
3. Determining of indicators resulting from these parameters;
4. Explaining the specific phenomenon measured by each indicator and its association with the relevant levels;
5. Developing a numerical database analysis;
6. Quantifying indicators.

As suggested by Porter, the outcome hierarchy is based on three main levels (each one divided into two sub-levels). A normalized scale with three positive and three negative values was created to facilitate the connection within the outcomes already set up. Cost (the equation’s denominator) refers to the total costs of the full cycle of care for the patient’s medical condition. In this study, only the parameters that were directly influenced by bringing in the nurse were considered. The Time-Driven Activity-Based Costing was identified as the best tool for cost assessment [22, 23]. The cost-related normalised index was built on a three-level scale as well, this time according to cost variation:

- Little variation, which does not change the budget,
- Relevant with respect to the budget,
- Variation that leads to a budget review.

**Fig. 1:**
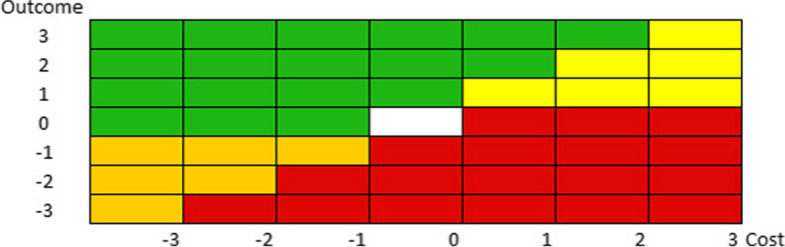
The matrix. The matrix is composed of zones marked by different colours. *White* – the value does not change; *Red* – the value is lower than the previous one; *Orange* – The value is lower than the previous one, but cost reduction is significant; *Yellow* – The value is higher, producing a similar increment on costs; *Green* – the value is higher than the previous one due to a significant increment in outcomes, with a low or no impact on costs

A measurement of the percentage change was needed to bring together multiple values into a single comprehensive indicator of outcome. Another criterion adopted was assigning weights the outcome levels concerned. This approach prompted the identification of the value of the intervention analyzed on the array. The results obtained by this study, outcomes as well as costs, were normalized to obtain an n-elements scale and provide the opportunity to replicate other measurements of value on the matrix (Fig. 1).

## Results

To better describe the considerable dataset of patients, general and specific parameters were considered. Gender was almost equally distributed among the two groups — 48.5% of the 2017 visits and 51.8% during 2019 regarded female patients — and the vast majority of the patients evaluated were affected by a rare skeletal disorder (95.9% for Group 1 and 99.1% for Group 2). The population dataset was composed of 274 pediatric patients, representing 45.4% of the entire dataset (40.6% for Group 1 and 49.1% for Group 2) (Table 1).

**Table 1:** The dataset organized according to specific criteria: by year, type of visit, gender, age and type of skeletal disorder of patients

	**2017**	**2019**
Number of visits	268	336
Number of follow-ups	137	201
Number of females	130	172
Number of paediatric patients	118	181
Number of skeletal disorders	18	16

### Evaluation of efficiency and effectiveness

#### Efficiency

The dataset was composed of 268 scheduled appointments carried out in 2017 (Group 1) plus 336 in 2019 (Group 2), making a total of 604 examinations evaluated. In 2017, 131 out of 268 were the first medical access, compared to 135 out of 336 in 2019, which can be easily explained by the increased number of visits between Group 1 and Group 2 due to follow-up visits. There was no significant difference in the number of diseases presented by patients referred to our service in 2019: 18 different rare dysplasias, compared to 16 rare skeletal disorders in 2017. The number of blood samples collected decreased from 32.8% (88 from 268 medical examinations) to 24.8% (83 samples collected from 336 visits). This difference is statistically significant, with a *p*-value of 0.036 (Table 2).

**Table 2:** A) line 1–7: Waiting Times in average days were analysed by the GLM Multivariate Analysis test, assessing separately first visits, follow-up, adult patients and children. B) line 8: Weighted Wait Times. C) line 9–12: lists withdrawal and order in IT records and clinical charts. D) line 13: project approved by an independent review committee

Variables	Group 1	Group 2	*p* Value
Waiting Times (average days)	49.56	36.24	*p* < 0.0005
Waiting Times adults	46.13	33.05	*p* < 0.0005
Waiting Times first visit – adults	35.09	23.32	*p* < 0.0005
Waiting Times follow-up – adults	56.49	40.66	*p* < 0.0005
Waiting Times paediatric patients	54.58	39.54	*p* < 0.0005
Waiting Times first visit—paediatric patients	42.5	26.02	*p* < 0.0005
Waiting Times follow-up—paediatric patients	66.4	47.27	*p* < 0.0005
Weighted Wait Times	18.49	10.79	*p* < 0.0005
Numbers of blood samples	83	88	*p* 0.036
Numbers of blood sampling	180	252	*p* 0.036
Order in clinical chart	242	312	*p* 0.231
Bias on IT records	35	14	*p* < 0.0005
Numbers of projects approved by EC	3	5	n.a

#### Effectiveness

To evaluate effectiveness, ad hoc appropriateness markers were identified and produced significant results. Using the Fisher Exact Test on orderliness and methodicalness in clinical charts, *p* = 0.231 was found with a value that increased from 90.3% (2017) to 93.1% (2019). A decreasing number of biases were found in recording personal information (i.e., date of birth, birthplace, address, etc.) in the hospital database (*p* < 0.0005). Thirty-five errors/typos out of 268 visits were reported (13.1%) in Group 1 versus 14 errors out of 335 in Group 2 (4.2%). The same significant results were found for studies involving day clinic activities submitted (and accepted) by the independent review committee, which increased from 3 in 2017 to 5 in 2019. However, some results were too fragmented to define a real trend. For a few effectiveness markers (appropriateness of special consultations and blood sample collections in patients without final diagnosis), the hypothesis to evaluate the appropriateness of special consultations was unenforceable due to an unexpected low number of visits. Finally, wait times (WT) were analysed through two statistical methods with different approaches. The first evaluation of wait times, by applying the GLM multivariate analysis test, was performed on all examinations in 2017 and 2019 and resulted in *p* < 0.0005, with an average of 49.56 days in Group 1, which decreased to 36.24 in Group 2, despite their being more examinations. The same test was applied to separately assess the first visit, follow-ups, adult patients, and children. The results were consistent, with significance values of *p* < 0.0005. All results are listed in detail in Table 2. To reduce the bias, wait times were evaluated with a more accurate approach. In fact, weighted wait times (WWT) were tested. This means that the number of patients was considered as fundamental (WWT = no. of days*100/no. patients). These results also confirmed with statistically significance (*p* < 0.0005) that the average WWT in 2017 (18.49 days/100 patients) was higher than in 2019 (10.79 days/100 patients).

### Perception of the service

The patient’s perception of service quality was evaluated by analyzing 34 out of 35 anonymous, semi-structured surveys. Just one interview was invalid because the patient described and evaluated hospitalization for surgical intervention instead of for the GDC. In 2019, 70.5% (24 surveys) of respondents reported positive changes compared to 2017, 2.95% (1 survey) revealed negative changes, and 26.45% answered “business as usual”. More specifically, four types of changes (patient reception, information provided, service availability wait times, and ambulatory care) were identified, with 71% of patients reporting shorter wait times and higher quality of ambulatory care, 63% reporting a positive trend in information provided (for clarity of the content and the supply of material), 58% liking that contacting the service was made simpler, and 54% stressing an increased quality of patients reception. Only 3% showed longer wait times.

### Cost-effectiveness results

**Fig. 2:**
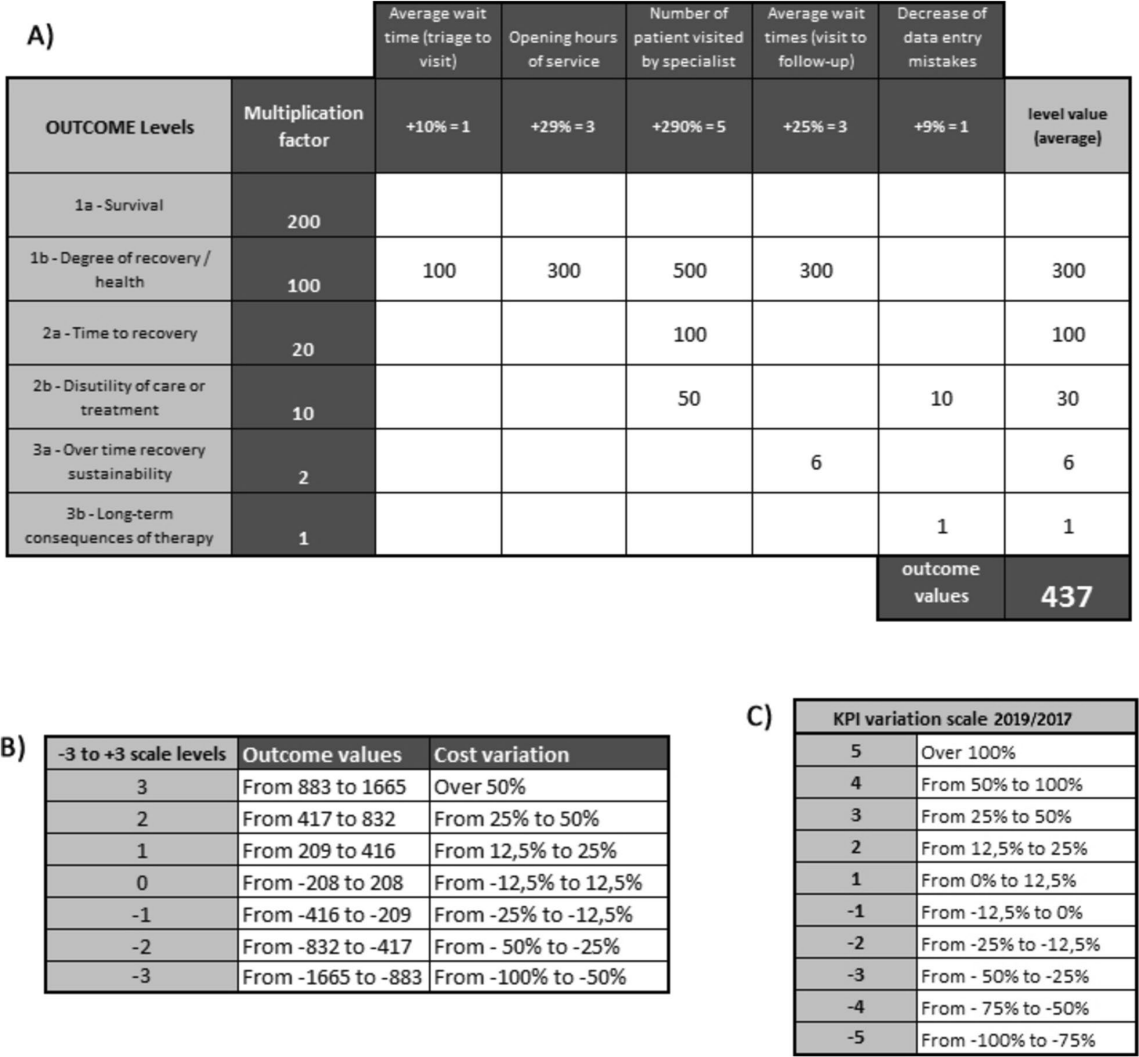
**A** *Outcome values and cost variation*: Costs (applying “Time driven Activity Based Costing” method) were compared “before” and “after” and a percentage variation was reported on a scale of -3 to 3. Data obtained during the evaluation are highlighted. **B** *Outcome values*: Calculation and distribution of percentage variations for each indicator. **C** *KPI Variation Scale*: Transformation of the percentage value in a scale of -5 to 5. KPI: Key Performance Indicators

Concerning the economic evaluation of this study, only the parameters that were directly influenced by the nurse’s presence were considered. The patient cycle is the cost object and includes the period from triage to the first follow-up, so as not to be influenced by the increased GDC activity over past years. Costs were calculated following the Time-Driven Activity-Based Costing method, estimating the time the nurse devoted. Then, this impact was compared before and after, which produced a percentage variation reported on a scale from -3 to 3 (Fig. 2).

The outcomes evaluation aimed at obtaining numerical data that could appropriately explain the collection of variations observed. The procedure is as follows:

1. Identification of the indicators subject to variation as per the analysis purpose.
2. Analysis of their detectability based on availability of the data. For non-detectable indicators, the importance and the strategies to make up for the lack of data were assessed.
3. Assignment of one of the 6 levels to each outcome, according to Porter’s hierarchy.
4. Calculation of the percentage variation for each indicator (Fig. 2B).
5. Transformation of the percentage value in a scale from -5 to 5 (Fig. 2C).
6. Multiplication for the univocal factor assigned to the level of the outcome related to indicator (second column of Fig. 2B).
7. Determination of the average value of the indicators relevant to each level, addition of the means and conversion of the result into a scale of -3 to 3 (Fig. 2A).

**Fig. 3:**
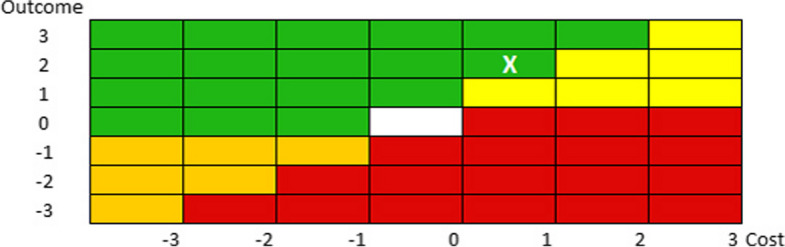
The matrix result. The white X indicates the position on the evaluation matrix derived from the cross-reference of costs and outcomes

The cross-reference of costs and outcomes enabled positioning on the evaluation matrix (Fig. 3).

## Discussion

To measure the impact of bringing a nurse into an existing GDC, a variety of efficiency and effectiveness indicators was identified —based on national accreditation requirements and the literature [21] — by a panel of multidisciplinary experts. As mentioned in the results, the number of clinical evaluations increased between 2017 and 2019, which would indicate greater efficiency of the medical genetics service, especially because of the rarity of the disease is a limiting factor. Moreover, the number of follow-up visits increased from 137 to 201, and similarly the first evaluations (131 and 135 respectively). Since “lost to follow up” is concrete problem in almost all medical fields, several scientific studies highlighted the pivotal role of nurses in continuity of care [24–26]. Taking this into consideration, as well as the known role of nurse in facilitating the communication bridge between patients and healthcare professionals in the hereditary oncology field [27, 28], the increased number of follow-up visits is a sign of the higher propensity of rare patients to continue clinical follow-up and may also be suggestive of better appropriateness of patient selection — performed by the nurse — for follow-ups.

The number of blood samples collected decreased considering the total number of visits, which could be mistakenly considered as a reduction in efficiency; however, it actually indicates a more accurate selection of patients eligible for molecular analyses due to a preliminary evaluation of cases by a nurse, thus demonstrating an overall increased efficiency. Data on blood sample collection from patients without final diagnosis were too small to give a significant result and, unfortunately, the hypothesis of evaluating the appropriateness of special consultations was unenforceable due to a very low number of visits. This patient dataset is an extremely small subgroup due to the ultra-rarity of the diseases treated.

To confirm the increased appropriateness and effectiveness, specific indicators (i.e., quality of data collection) were identified. For capturing clinical data, Fisher’s Exact Test clearly suggests that information is more available and better organized. In addition, considering the collection of patient personal data, a very significant decrease in bias (*p* < 0.0005) was detected due to an accurate checking of all personal information in the hospital database, thus suggesting the value of nurses in the data capturing process.

The time that elapsed between the date of reservation to the date of examination, otherwise known as wait times and weighted wait times, reflects the rationing and prioritizing of health services and is a known issue in many biomedical fields [29, 30]. Additionally, it represents an indicator collected by the Public Relations Office to evaluate the healthcare performance of the institute, as well as parameters highlighted among the national and regional specific accreditation requirements [20]. The present results highly emphasize the role of nurses in GDCs. In fact, patient categories (adults and children) and both types of examinations (first visits and follow-ups) showed a highly statistically significant decrease in wait times from 2017 to 2019, also considering the increased number of visits.

Irrespective of specific knowledge, genetics training, role in genetic counselling, or specific medical treatments, the presence of a nurse in a medical genetics service ensured the evaluation of markers of efficiency and effectiveness and produced improvements in healthcare, as per the literature related to orthopaedic department [31]. Not only did efficiency and effectiveness significantly increase after brining a nurse into the GDC, but patient perception of service quality also followed the same trend.

Capturing patient satisfaction using self-reported surveys is considered a reliable method by the scientific community [32, 33]. The anonymous, semi-structured surveys showed a marked improvement in outpatient management on all evaluated indicators. More than four fifths of the interviews showed one or more positive changes, distributed similarly between the four options proposed. Of the remaining one fifth, 90% did not indicate any changes, and only one interview showed a negative impact, indicating an increase in wait times.

The value in healthcare is affected by hundreds of needs and expectations, sometimes diverging or even conflicting and incompatible, due to the presence of a variety of stakeholders [34–36]. In addition to the detected positive impact on efficiency, effectiveness and patient perception of brining a nurse into the GDC, the present study also aimed to measure the efforts and evaluate the economic impact of this implementation. To this end, several indicators were identified, and their detectability was assessed in terms of variation. Then, the outcomes were evaluated in six levels following Porter’s methodology adapted to the specific context [22]. This analysis highlighted the impact, measured as patient-centric care, on outcomes and costs as shown by the matrix comparing the results for the two years. In fact, a greater increase in overall outcomes compared to costs was measured in 2019 than in 2017.

Although this study analyzed bringing a nurse into a GDC and the resulting impact on several indicators, some limitations need to be addressed. First, while the multidisciplinary panel identified several indicators, the ultra-rarity of some diseases impacted the amount of data available, so the evaluation of appropriateness of special consultations was unenforceable. Second, the pencil-and-paper survey could have been more detailed, asking additional questions, giving us the opportunity to have a more comprehensive overview of patient’s perception.

## Conclusion

In conclusion, the present study, collecting data and evaluating multiple indicators and measurements, highlights that bringing a nurse into a medical genetics service produced positive results. In fact, we clearly highlight a better patient management – captured by patients’ perspective as well as deduced from efficacy and effectiveness indicators—an increased quality of service, impacting also on the National Health System, and, on top of this, a greater increase in overall outcomes compared to costs.

### Supplementary Information

**Additional file 1.** **Supplementary Material 1.** Italian Survey for patient's perception of service quality.

## Data Availability

The datasets used and/or analysed during the current study are available from the corresponding author on reasonable request.

## Abbreviations

GDC: Genetic day clinic
GLM: General linear model
WT: Wait times
WWT: Weighted wait times
