# Supplementary material for: Cost-effectiveness of bringing a nurse into an Italian genetic day clinic: a before and after study
Source: BMC Health Serv Res. 2023 Nov 20;23:1278. doi: 10.1186/s12913-023-10238-8 (PMC10662525; doi:10.1186/s12913-023-10238-8)
Supplement: Supplementary file 1 — Additional file 1. Supplementary Material 1. Italian Survey for patient's perception of service quality. [file 12913_2023_10238_MOESM1_ESM.pdf]

INTERVISTA SEMI STRUTTURATA

1. Riportandola indietro negli anni, in particolare a quando ha iniziato il percorso clinico presso il nostro ambulatorio ha notato dei cambiamenti nella organizzazione rispetto alla situazione odierna?
   - SI ☐
   - NO ☐

   - Se ha risposto SI, quali:

   Elementi positivi

…………………………………………………………………………………………………

…………………………………………………………………………………………………

…………………………………………………………………………………………………

…………………………………………………………………………………………………

   Elementi negativi

…………………………………………………………………………………………………

…………………………………………………………………………………………………

…………………………………………………………………………………………………

…………………………………………………………………………………………………

Istituto Ortopedico Rizzoli
Sede legale Via Di Barbiano, 1/10 - 40136 Bologna
Ospedale Via G. C. Pupilli, 1 - 40136 Bologna
Centro di Ricerca Via Di Barbiano, 1/10 - 40136 Bologna
Tel. 051/6366111 - **Fax** 051/580453
Sito web: www.ior.it
Codice Fiscale e Partita IVA 00302030374

2. In particolare, pensando alle visite fatte prima del 2018 e dal 2019, ha notato cambiamenti:

- Nell'accoglienza in ambulatorio SI ☐ NO ☐

  i. se sì quali……………………………………………………………………….

  ………………………………………………………………………………..

- Nelle informazioni ricevute SI ☐ NO ☐

  i. se sì quali……………………………………………………………………….

  ………………………………………………………………………………...

- Nella possibilità di contattare il servizio SI ☐ NO ☐

  i. se sì quali……………………………………………………………………….

  ………………………………………………………………………………..

- Attesa nella sala d'aspetto SI ☐ NO ☐

  i. se sì quali………………………………………………………………………

  ……………………………………………………………………………….

Se vuole aggiungere altri commenti……………………………………………………………...

…………………………………………………………………………………………………

…………………………………………………………………………………………………

…………………………………………………………………………………………………

…………………………………………………………………………………………………

Grazie per la collaborazione
Ambulatorio di Genetica Medica
